# Evaluation of Active Transition, a Website-Delivered Physical Activity Intervention for University Students: Pilot Study

**DOI:** 10.2196/resprot.2099

**Published:** 2013-05-06

**Authors:** Matthew Kwan, Guy Faulkner, Steven Bray

**Affiliations:** ^1^Department of Family MedicineFaculty of Health SciencesMcMaster UniversityHamilton, ONCanada; ^2^Exercise Psychology UnitFaculty of Kinesiology and Physical EducationUniversity of TorontoToronto, ONCanada; ^3^Department of KinesiologyFaculty of ScienceMcMaster UniversityHamilton, ONCanada

**Keywords:** physical activity, efficacy trial, Internet-based intervention, university students

## Abstract

**Background:**

While physical activity in individuals tends to decline steadily with age, there are certain periods where this decline occurs more rapidly, such as during early adulthood. Interventions aimed at attenuating the declines in physical activity during this transition period appear warranted.

**Objective:**

The purpose of the study was to test the feasibility and efficacy of a theoretically informed, website-delivered physical activity intervention aimed at students entering university.

**Methods:**

Using a quasi-experimental design, 65 participants (44 females; mean age 18.51, SD 0.91) were assigned to either an intervention (receiving website access plus weekly prompts) or comparison condition (receiving unprompted website access only), completing questionnaires at baseline and follow-up 8 weeks later. The intervention website, “Active Transition”, was specifically designed to target students’ physical activity cognitions and self-regulatory skills.

**Results:**

Intervention usage was low, with only 47% (18/38) of participants assigned to the intervention condition logging into the website 2 or more times. Among the broader student sample, there were significant declines in students’ physical activity behaviors (*F*_1,63_=18.10, *P*<.001), attitudes (*F*_1,62_=55.19, *P*<.001), and perceived behavioral control (*F*_1,62_ =17.56, *P*<.001). In comparisons between intervention users (29/65, individuals logging in 2 or more times) and non-users (36/65*,* individuals logging in once or not at all), there was a significant interaction effect for intervention usage and time on perceived behavioral control (*F*_1,62_=5.13, *P=*.03).

**Conclusions:**

Poor intervention usage suggests that future efforts need to incorporate innovative strategies to increase intervention uptake and better engage the student population. The findings, however, suggest that a website-delivered intervention aimed at this critical life stage may have positive impact on students’ physical activity cognitions. Future studies with more rigorous sampling designs are required.

## Introduction

Despite many known health benefits of physical activity, the majority of the Western world does not accrue recommended amounts of moderate-to-vigorous physical activity (MVPA) [1,2]. Given that physical inactivity is a pervasive problem across the general population, concerted efforts are required to develop interventions aimed at specific life stages [3,4]. While most research has focused on increasing participation, little work has been aimed at preventing physical activity declines. Trends across the lifespan show children and youth being the most active segment in the population; however, accelerated erosions in physical activity are evident as this youth population moves toward early adulthood [5,6]. More recently, a number of studies have found the transition from high school to college/university being a period in which young adults are particularly susceptible to significant declines in physical activity [7-9]. Approximately one-third of the young adults that were sufficiently active during high school, become insufficiently active during their first year at university [8]. These declines in physical activity might be largely unexpected as most students enter university with positive attitudes toward physical activity and strong intentions to be active [9,10]. Given that there is potential for exposing university students to sustained health messaging through already established knowledge exchange methods and messengers, and subsidized physical activity facilities, programs, and staffing, the post-secondary environment should be an ideal setting of intervention to attenuate declines in physical activity in contrast to interventions seeking to increase physical activity.

Few attempts, however, have been made to address population-specific perturbations in social (eg, peer influence) and environmental (eg, moving away from home) conditions. To the best of our knowledge, only one intervention study has explicitly targeted students’ transition into college and university. Bray and colleagues [11] developed a tailored physical activity guide targeting students entering first year college and university, and the results of the intervention were positive. Compared to students who were given a standard physical activity guide or no guide, those receiving a tailored activity guide were engaging in significantly more MVPA per week during the 6-week intervention period. The caveat, however, was that students who received the tailored activity guide still exhibited significant declines in physical activity upon entry into university. This suggested that further effort is required to develop effective interventions for this population. In particular, although print-based material is low in cost and easy to mass distribute, the Internet is becoming increasingly popular for health behavior change interventions [12-14], and although its effectiveness has varied [15], it is advantageous as information can be delivered in real time and accessed by users at their convenience. Intuitively, the Internet would be an appropriate platform within the context of the university setting. Recent research also provided empirical support, as the Internet is the most frequently used source from which university students gain health-related information [16], and the Internet has been identified as the preferred delivery vehicle for any potential physical activity intervention [10]. The purpose of this investigation was to pilot the feasibility and efficacy of a theoretically informed, website-delivered physical activity intervention called “Active Transition”. Specifically, the intervention aimed at attenuating the declines in physical activity behaviors among students as they transition into university.

## Methods

### Selection and Recruitment of Participants

The current study was a quasi-experimental trial using stratified cluster randomization to recruit participants. A total of 4 floors in residence were asked to take part in this pilot study. There were 2 campus residence buildings that participated, each having 1 floor randomly (by toss of a coin) assigned to the intervention condition, and 1 floor selected to the comparison condition. Early in the fall semester, 198 eligible students living on the selected floors were invited to participate. Written consent was obtained from 146 potential participants. These students were sent a link to the baseline questionnaire, which included measures of demographic characteristics, psychosocial variables of attitudes, subjective norms, perceived behavioral control and intentions, and physical activity behaviors. Baseline data was obtained from 91 (59 female) students, with most students in their first year of study. Following the pilot 6-week intervention (described in detail below), participants that completed the baseline questionnaire were sent a link to a follow-up questionnaire with the same measures of physical activity cognitions and behavior. Further attrition resulted in a final sample of 65 students (44 female; mean age 18.51, SD 0.92) completing both baseline and follow-up questionnaires. The study protocol was approved by the Research Ethics Board at the University of Toronto.

### Study Conditions

For pragmatic reasons, and given the pilot nature of the study, a true control group was not included. A minimal-contact condition or comparison condition was used instead, where students were provided access to, but were not prompted to use, the intervention website. Given that the effectiveness of Internet-based interventions are greatly enhanced with the use of additional methods of participant interactions such as email messages [14], participants in the intervention condition were sent weekly emails prompting students to access the intervention website. The emails provided participants with a synopsis highlighting a weekly intervention topic for each of the 6 weeks. For instance, the topic for week 2 of the intervention was focused on the many student-specific benefits of maintaining a physically active lifestyle. To illustrate, students were sent the following email:

Hope you’re having a great week… Did you know? Being physically active can help you obtain better grades. Physical activity can also give you more energy, allow you to concentrate better, and get you a better night’s rest! Find out more about how physical activity can help you with your studies and more.

An external link accompanied the email message that linked participants to the website with more details about the student-specific benefits of being physically active.

### Active Transition

Hosted within a university portal (Blackboard), Active Transition was a password-controlled website developed to be an informational forum specifically targeting psychosocial mediating variables based on Ajzen’s [17] theory of planned behavior (TPB), and educating students around self-regulating/self-monitoring techniques. Findings from a recent review reported that Web-based TPB interventions are more effective than other theory-based interventions (eg, social cognitive theory, transtheoretical model) [14], and that control theory techniques (eg, goal setting, action planning) are most effective for changing physical activity behaviors [18]. Designed to be 6 weeks in length, Active Transition was a passive intervention, delivering weekly topics on behavioral, normative, control beliefs in physical activity, as well as goal-setting, action planning, relapse prevention, and behavioral maintenance.

**Figure 1:**
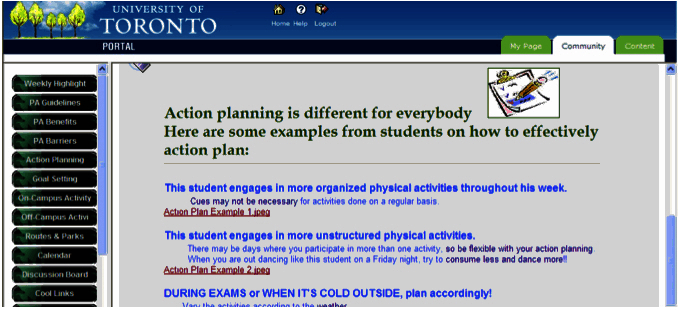
Screenshot of the intervention topic on action planning.

**Figure 2:**
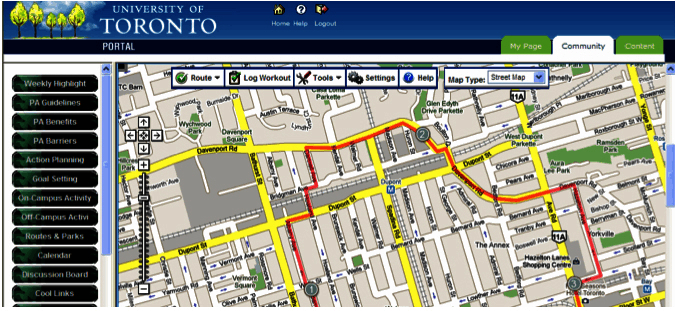
Screenshot of running map as example of intervention resources.

The first 2 weeks of the intervention targeted students’ motivation (ie, attitudes, perceptions of control), including student-specific benefits of being physically active (eg, improving concentration, helping with social life), and coping strategies to deal with salient barriers that students typically face during their transition into university. In an attempt to bridge the gap between students’ intentions and subsequent behaviors [9], topics for week 3 and 4 focused on behavioral modification techniques. The intervention material included information on how to effectively set goals, as well as providing sample schedules of how to action plan (Figure 1). Action planning and goal setting are two strategies that have been found to be effective in translating people’s intention into behaviors [19,20]. The topics for the final 2 weeks of the intervention focused on relapse prevention and behavioral maintenance, encouraging students to maintain behaviors despite the normal disruptions that occur while at university. In addition to the highlighted topics, the website included additional resources for students, including a discussion board to facilitate social networking (eg, finding other people with similar interests), links to available leagues and facilities on or nearby campus, mapped out running/biking routes (Figure 2), as well as the opportunity to contact a physical activity expert. Prior to the intervention, Active Transition was pre-tested with students and physical activity experts. A few minor changes were made regarding the text and material; however, there was consensus that the intervention was both acceptable and user-friendly [21].

### Measures

#### Moderate-to-Vigorous Activity

MVPA was measured using the 2003 Behavior Risk Factor Surveillance System (BRFSS; CDC, 2003 [22]). Participants reported the *average number of sessions* as well as the *average duration* of both moderate and vigorous physical activity engaged in per week. Example questions used to collect this information included (1) in a usual week, how many days do you do vigorous activities (such as running, aerobics, hockey, squash) for at least 10 minutes at a time that cause large increases in breathing or heart rate? and (2) On days that you do vigorous activities for at least 10 minutes at a time, how much total time per day do you spend doing these activities? MVPA was computed by adding the products of the average weekly frequency and duration for both moderate and vigorous intensity activities. Consistent with previous research on students’ transition into university [8,9], an 8-month recall was used to capture average MVPA prior to the intervention and 6-week recall was used at the follow-up measure to capture average MVPA during the intervention period.

#### Physical Activity Cognitions

Social cognitive variables comprised of TPB measures that were developed and used in a previous study [9,23]. For the purposes of clarity and consistency, each measure used the common reference of being “physically active” (defined as engaging in MVPA on most days of the week for at least 30 minutes per day). Multi-item measures were summed and averaged; reliability of scales were all within acceptable range (alpha=.76 to .91).

#### Attitudes

Rated on a 7-point Likert scale, 6 items were used to measure attitudes. Two items captured the instrumental component (ie, being physically active is harmful/beneficial and useless/useful), 3 represented the experiential component (ie, enjoyable/unenjoyable, pleasant/unpleasant, and fun/boring), and a good-bad scale.

#### Subjective Norms

A single item was used to reflect subjective norms, asking: important people to me think I should be physically active. Participants were required to rate each item on a 7-point Likert scale (1= strongly disagree, 7= strongly agree).

#### Perceived Behavioral Control

To measure PBC, 6 items were used. Three questions assessed controllability and 3 questions assessed self-efficacy. For example, questions pertaining to controllability included, “how much control do you have to be physically active?” (1=extreme lack of control, 7=extreme control), and questions assessing self-efficacy included, “how confident are you that you can be physically active?” (1=extremely unconfident, 7=extremely confident).

#### Physical Activity Intentions

Three items were used to measure participants’ intentions to be physically active (1=strongly disagree, 7=strongly agree), asking, “I intend, I will try, and it is my desire to be physically active”.

## Results

### Process Evaluation

**Figure 3:**
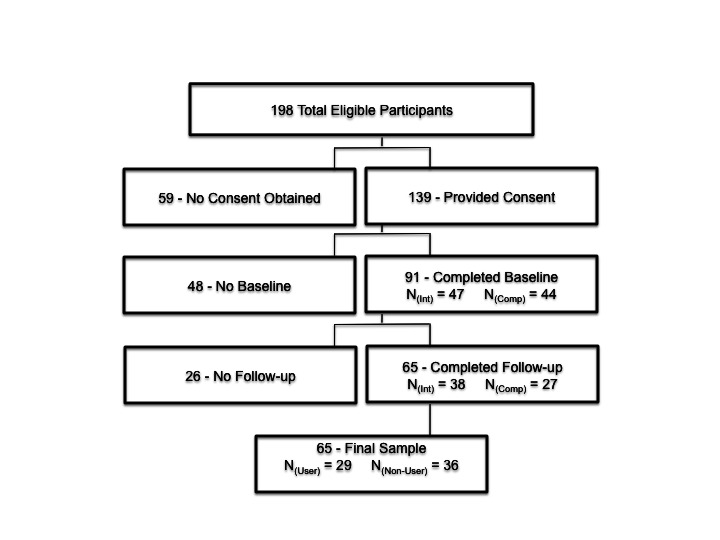
Detailed breakdown of participant recruitment.

To examine the feasibility of the website-delivered intervention, study compliance was first examined. Among a total of 198 eligible participants living in the selected residences, 65 of the students completed both baseline and follow-up questionnaires. Initially, 74% (139/198) of all the eligible students had provided written consent, expressing interest in the physical activity intervention and participating in the study. Baseline data, however, was obtained from only 91 (59 females) of those students, representing a 62% response rate; and of the 91 participants that completed the baseline questionnaire, 65 completed the follow-up questionnaire (44 females), representing a 71% (65/91) retention rate. One-way ANOVAs revealed no significant differences between adherers and dropouts for baseline physical activity levels (*F*_1,89_=1.67, *P*=.17), or on any of the physical activity cognition measures (*P’s* >.05); thus the final sample of 65 participants was available for subsequent analyses. A comprehensive breakdown of participant responses is shown in Figure 3.

To determine the level of engagement that participants had with the intervention, usage data (the number of times participants logged into the intervention website) was also examined. Importantly, it uncovered that 41% of the participants assigned to the comparison condition were actually users of the intervention (11/27, defined as logging into the website 2 or more times), and 53% in the intervention condition were non-users (20/38, defined as logging in once or not at all). More broadly, usage results indicated that compliance to the intervention was low, with only 6% of participants (4/65) entering the website on an average of one time per week (ie, 6 or more occasions), and only 45% of participants (29/65) logging onto the intervention website on more than 2 occasions. Similarly there were only 5 participants that used the discussion board during the intervention period, and only 1 student had contacted the physical activity expert. Given that this was a pilot study that resulted in unexpected usage within both study conditions, subsequent comparisons in physical activity cognitions and behaviors are made between intervention users (29/65) and intervention non-users (36/65).

### Changes in Physical Activity Cognitions and Behaviors

The descriptive statistics showing mean scores (SD) for the users and non-users at baseline and follow-up are presented in Table 1. Results of an initial one-way ANOVA found no significant differences in baseline MVPA between users and non-users of the intervention F_1,63_=0.13, *P*=.72). Differences between groups and over time for each of the measured variables were evaluated using the following equation: 2 (time: baseline/follow-up) X 2 (usage: user/non-user), with repeated measures ANOVAs treating the first factor as a within-subjects variable and the second factor as a between subjects variable. Results revealed significant main effects for time, in MVPA (*F*_1,63_=18.1, *P*<.001, η_p_^2^=.22), attitudes (*F*_1,62_ =55.19, *P*<.001, η_p_^2^=.47), and perceived behavioral control (*F*_1,62_=17.56, *P*<.001), η_p_^2^= .22). The main effect of time for intentions (*F*_1,62_=2.91, *P*=.08, η_p_^2^=.06) also approached significance. Main effects for usage conditions were all non-significant (*P*’s>.05).

A significant interaction effect was observed between usage and time on perceived behavioral control (*F*_1,62_ =5.13, *P=*.03, η_p_^2^=.08), indicating that intervention users maintained higher levels of perceived control over time, while steeper declines in these perceptions occurred among non-users from baseline to follow-up. While the interaction effects were non-significant between usage and time on attitudes (*F*_1,62_=2.02, *P*=.16, η_p_^2^=.03) and usage and time on intentions (*F*_1,62_=1.97, *P*=.16, η_p_^2^=.03), mean scores suggest that intervention users were also able to better maintain positive attitudes and higher physical activity intentions while non-users showed greater negative changes in these perceptions over time. The interaction effect between intervention usage and time on physical activity (*F*_1,63_=1.54, *P*=.22, η_p_^2^=.03) was not significant. Additional analyses, however, uncovered significant differences in the proportion of students exhibiting declines in their MVPA during the intervention period comparing intervention users and non-users (*χ* ^2^_1_=6.11, *P=*.01, Cohen’s *d*=.64). The results found fewer intervention users (15/29, 48%) declined in their MVPA scores from baseline to follow-up compared to non-users (8/36, 78%). Findings did not differ when intention-to-treat analyses were conducted, treating dropouts as non-users.

**Table 1:** Descriptive statistics of physical activity behaviors and cognitions by study condition.

			Study conditions	
Variables		Full sample N=65 Mean (SD)	Intervention users^a^ n=29 Mean (SD)	Non-users^b^ n=36 Mean (SD)
**Baseline**				
	MVPA	595.00 (424.41)	594.47 (443.49)	595.74 (404.34)
	Attitude	5.52 (0.93)	5.52 (0.84)	5.52 (1.01)
	Subjective norm	5.25 (1.59)	5.24 (1.38)	5.25 (1.52)
	PBC	5.44 (0.96)	5.46 (0.86)	5.43 (1.04)
	Intentions	6.01 (0.96)	5.99 (0.86)	6.03 (1.04)
**Follow-up**				
	MVPA	366.96 (341.35)	393.47 (352.18)	329.63 (328.37)
	Attitude	4.39 (0.86)	4.63 (0.69)	4.18 (0.94)
	Subjective norm	5.38 (1.68)	5.21 (1.38)	5.25 (1.76)
	PBC	4.90 (1.10)	5.21 (0.71)	4.63 (1.30)
	Intentions	5.79 (1.28)	5.94 (0.77)	5.66 (1.59)
	Barriers self-efficacy	5.49 (2.52)	5.61 (2.65)	5.32 (2.38)

^a^Intervention users were participants in the intervention condition that logged in 2 or more times

^b^Non-users were participants in the comparison condition that logged in 1 or less times.

## Discussion

### Principal Findings and Further Research

Overall, the results of this pilot study were largely mixed. Positive results found 75% (139/198) of the students living in the selected residences had initially expressed interest in the study (ie, being a part of a physical activity intervention), and that implementation of the website-delivered physical activity intervention on campus is feasible and of interest to students. Participant engagement, however, was highly problematic, as response, retention, and compliance rates were all low. In particular, it was alarming that only 45% (14/38) of the participants in the intervention condition were considered users of the intervention, despite a liberal categorization of usage (ie, considered users if logged in 2 or more times over 6 weeks). At minimum, participants should have been accessing the intervention on a weekly basis. The low usage appears consistent with the notion that students may be generally ambivalent about their physical activity levels [10]. However, with intervention usage, there is some evidence that Active Transition may be helpful in attenuating some declines in students’ physical activity cognitions and behaviors.

Adherence to intervention protocols is essential for physical activity interventions to be successful [24], and our results clearly demonstrated the need to identify innovative strategies to both engage participants and maintain their interest over the duration of the intervention. The development of Active Transition was constrained by limited financial resources. With adequate funding, future interventions with appropriate resourcing should incorporate other Internet-based tools, such as social media, social networking, and/or enhanced visual prompts (ie, video-messaging), and increase participant interaction (eg, providing students with immediate feedback) [25,26]. In an effort to better engage the student population, Active Transition for example, can be augmented to incorporate elements of social media (ie, Facebook, Twitter), and/or integrate the use of short message service (SMS) and photo/video text messaging. There is empirical evidence to suggest that intervention effects are markedly improved if it is theory-based and integrated with personalized contact [13,14]. Given that technology has profoundly changed the way information is being delivered, and how people connect with one another, it appears imperative that attempts are made to integrate these emerging technologies with intervention efforts aimed at behavior change. However, it may also be necessary to first better understand what appeals to students, and how they would want technological tools incorporated to help them facilitate greater physical activity participation.

Consistent with the research literature [7-9], there were significant declines in MPVA among the university students between baseline and follow-up, both for intervention users and non-users. Despite a non-significant interaction between average MPVA and usage, a significantly greater proportion of intervention users were able to maintain or even increase their physical activity behaviors. It is noteworthy that users of the intervention reported engaging in 30 minutes of MVPA more each week during the 6-week intervention period, and if maintained over time, this could have some clinical significance. One plausible reason, however, for why the intervention did not have a statistically significant interaction effect on the mean physical activity scores is because Active Transition specifically targeted MVPA declines through psychosocial mediators (ie, TPB variables). MacKinnon [27] suggested that if the intervention is specifically developed to target social cognition variables, it is very likely that there would be a delayed effect on the behavioral outcome. In other words, the length of the intervention may have been too short to see meaningful differences in behavior. It is necessary to ensure that future implementation of the intervention targets these proposed mediators with a higher fidelity [28].

Findings from this pilot also uncovered substantial decreases in students’ attitudes, perception of control, and physical activity intentions over the course of the first semester. Given that most participants were first-year students, decreases in physical activity cognitions may be a direct reflection of the adaptations required for students entering a new environment, consisting of more barriers to physical activity compared to at high school [10,29], entering a volatile period requiring constant changes and corresponding adjustments. There was evidence to suggest that intervention users were better able to maintain their perceptions of control. Maintaining strong perceived control and intentions towards activity is critical, as they are considered to be the most proximal determinants of behavior [16]. Larger efficacy trials are needed to determine the extent to which perceptions of control are maintained over the course of a calendar year at university. Future work is also required to replicate these findings, and to determine whether the attenuations in students’ physical activity cognitions can lead to significant attenuations in the declines in physical activity typically seen during students’ transition into university.

### Study Limitations

There are several important limitations to acknowledge. First, a self-report measure was used to assess physical activity, and such measures are susceptible to recall errors and social desirability bias. Second, the study was limited by a small sample size. Given that the purpose of the study was to pilot the feasibility and efficacy of a newly developed intervention, power calculations were not conducted a priori. However, a post-hoc power calculation was conducted, confirming that we were indeed underpowered (1-ß_err prob_=0.69). To be sufficiently powered, the sample size would require 43 participants in each group. Third, the study design excluded a true control group, resulting in unanticipated usage for some participants initially assigned to the comparison condition. Lastly, it should be acknowledged that the study design had a relatively short follow-up period. Without a subsequent follow-up, it is unknown whether the declines in physical activity cognitions are sustained throughout the academic year.

### Conclusions

Notwithstanding the notable limitations, this was the first theory-based website-delivered physical activity intervention aimed at students entering university. Although the institutional portal provided a platform for Active Transition that was potentially advantageous in terms of student access (eg, approximately 70,000 students have access to it at this institution), and its high potential for adoption and diffusion by other colleges or universities, there were major issues with low usage of the intervention. Future work must better target student engagement. Modifications to future interventions should utilize and incorporate other technological and interactive tools that motivate students to continually engage with the intervention. Physical activity decline continues to be problematic during students’ transition into early adulthood, and research must continue to develop innovative strategies for encouraging students to maintain a physically active lifestyle that can be sustained through university and beyond.

## Abbreviations

BRFSS: Behavior Risk Factor Surveillance System
MVPA: moderate-to-vigorous physical activity
SMS: short message service
TPB: theory of planned behavior

## References

[1] Dishman RK, Washburn RA, Heath GW (2004). Physical Activity Epidemiology.

[2] Kimm SY, Glynn NW, Obarzanek E, Kriska AM, Daniels SR, Barton BA, Liu K (2005). Relation between the changes in physical activity and body-mass index during adolescence: a multicentre longitudinal study. The Lancet.

[3] King AC (1994). Community and public health approaches to the promotion of physical activity. Med Sci Sports Exerc.

[4] Sparling PB, Owen N, Lambert EV, Haskell WL (2000). Promoting physical activity: the new imperative for public health. Health Educ Res.

[5] Caspersen CJ, Pereira MA, Curran KM (2000). Changes in physical activity patterns in the United States, by sex and cross-sectional age. Med Sci Sports Exerc.

[6] Gordon-Larsen P, Nelson MC, Popkin BM (2004). Longitudinal physical activity and sedentary behavior trends: adolescence to adulthood. Am J Prev Med.

[7] Kwan MY, Cairney J, Faulkner GE, Pullenayegum EE (2012). Physical activity and other health-risk behaviors during the transition into early adulthood: a longitudinal cohort study. Am J Prev Med.

[8] Bray SR, Born HA (2004). Transition to university and vigorous physical activity: implications for health and psychological well-being. J Am Coll Health.

[9] Wing Kwan MY, Bray SR, Martin Ginis KA (2009). Predicting physical activity of first-year university students: an application of the theory of planned behavior. J Am Coll Health.

[10] Kwan MYW, Faulkner G (2011). Perceptions and barriers to physical activity during the transition to university. Am J Health Stud.

[11] Bray SR, Beauchamp MR, Latimer AE, Hoar SD, Shields CA, Bruner MW (2011). Effects of a print-mediated intervention on physical activity during transition to the first year of university. Behav Med.

[12] Vandelanotte C, Spathonis KM, Eakin EG, Owen N (2007). Website-delivered physical activity interventions a review of the literature. Am J Prev Med.

[13] van den Berg MH, Schoones JW, Vliet Vlieland TP (2007). Internet-based physical activity interventions: a systematic review of the literature. J Med Internet Res.

[14] Webb TL, Joseph J, Yardley L, Michie S (2010). Using the internet to promote health behavior change: a systematic review and meta-analysis of the impact of theoretical basis, use of behavior change techniques, and mode of delivery on efficacy. J Med Internet Res.

[15] Davies CA, Spence JC, Vandelanotte C, Caperchione CM, Mummery WK (2012). Meta-analysis of Internet-delivered interventions to increase physical activity levels. Int J Behav Nutr Phys Act.

[16] Kwan MY, Arbour-Nicitopoulos KP, Lowe D, Taman S, Faulkner GE (2010). Student reception, sources, and believability of health-related information. J Am Coll Health.

[17] Ajzen I (1991). The theory of planned behavior. Organizational Behavior and Human Decision Processes.

[18] Michie S, Abraham C, Whittington C, McAteer J, Gupta S (2009). Effective techniques in healthy eating and physical activity interventions: a meta-regression. Health Psychol.

[19] Gollwitzer PM (1999). Implementation intentions: Strong effects of simple plans. American Psychologist.

[20] Smith JA, Hauenstein NMA, Buchanan LB (1996). Goal Setting and Exercise Performance. Human Performance.

[21] Kwan MYW, Faulkner G (2010). Active Transition: pre-testing a website-delivered physical activity intervention.

[22] Centers for Disease Control and Prevention (1998). 1996 BRFSS summary prevalence report.

[23] Ajzen I (2007). Theory of planned behavior.

[24] Heesch KC, Mâsse LC, Dunn AL, Frankowski RF, Mullen PD (2003). Does adherence to a lifestyle physical activity intervention predict changes in physical activity?. J Behav Med.

[25] Rhodes RE, Pfaeffli LA (2010). Mediators of physical activity behaviour change among adult non-clinical populations: a review update. Int J Behav Nutr Phys Act.

[26] Fenner Y, Garland SM, Moore EE, Jayasinghe Y, Fletcher A, Tabrizi SN, Gunasekaran B, Wark JD (2012). Web-based recruiting for health research using a social networking site: an exploratory study. J Med Internet Res.

[27] Whittaker R, Dorey E, Bramley D, Bullen C, Denny S, Elley CR, Maddison R, McRobbie H, Parag V, Rodgers A, Salmon P (2011). A theory-based video messaging mobile phone intervention for smoking cessation: randomized controlled trial. J Med Internet Res.

[28] MacKinnon DP (2008). Mediation Moderation. Introduction to statistical mediation analysis.

[29] Gyurcsik NC, Spink KS, Bray SR, Chad K, Kwan M (2006). An ecologically based examination of barriers to physical activity in students from grade seven through first-year university. J Adolesc Health.

